# Insights Into the Factors Influencing Student Motivation in Augmented Reality Learning Experiences in Vocational Education and Training

**DOI:** 10.3389/fpsyg.2018.01486

**Published:** 2018-08-21

**Authors:** Jorge Bacca, Silvia Baldiris, Ramon Fabregat, Kinshuk

**Affiliations:** ^1^Institute of Informatics and Applications, University of Girona, Girona, Spain; ^2^Direction of Research, Innovation and Social Projection, Fundación Universitaria Tecnológico Comfenalco, Cartagena, Colombia; ^3^Universidad Internacional de la Rioja, Logroño, Spain; ^4^College of Information, University of North Texas, Denton, TX, United States

**Keywords:** augmented reality, ARCS, learning experiences, motivation, vocational education and training

## Abstract

Research on Augmented Reality (AR) in education has demonstrated that AR applications designed with diverse components boost student motivation in educational settings. However, most of the research conducted to date, does not define exactly what those components are and how these components positively affect student motivation. This study, therefore, attempts to identify some of the components that positively affect student motivation in mobile AR learning experiences to contribute to the design and development of motivational AR learning experiences for the Vocational Education and Training (VET) level of education. To identify these components, a research model constructed from the literature was empirically validated with data obtained from two sources: 35 students from four VET institutes interacting with an AR application for learning for a period of 20 days, and a self-report measure obtained from the Instructional Materials Motivation Survey (IMMS). We found that the following variables: use of scaffolding, real-time feedback, degree of success, time on-task and learning outcomes are positively correlated with the four dimensions of the ARCS model of motivation: Attention, Relevance, Confidence, and Satisfaction. Implications of these results are also described.

## Introduction

Augmented Reality (AR) is rapidly evolving (Kim et al., 2016) as is research on AR in education (Santos et al., 2014; Saidin et al., 2015). The increasing interest in using AR in education has led to the creation of AR learning experiences (ARLEs), i.e., learning experiences supported by AR (Santos et al., 2014) and many ARLEs have been created for almost all levels of education from early childhood education through to higher education. Consequently, the many advantages of and limitations to, challenges and opportunities for this technology in education have been reported in the literature. Two of the most relevant advantages of AR applications in education are: increased learning outcomes and increased motivation (Chiang et al., 2014; Radu, 2014). Therefore, if AR applications boost student motivation, the AR applications have been designed with components that positively affect student motivation when students interact with these components during an ARLE. However, most of the research into student motivation in ARLE does not clearly identify which are the components of AR applications that may contribute to increase student motivation and does not explain how and why motivation is increased. Thus, further research is needed on student motivation to have a better understanding of the advantages of AR in education (Akçayır and Akçayır, 2017).

Consequently, this study aims to provide some insights into the components of AR applications that increase student motivation to contribute to the design and development of AR applications that effectively increase motivation (that we call motivational mobile ARLEs). In this paper we focused on mobile ARLEs and the research questions that drive this study are:

(1) Which are the components of mobile AR applications that positively affect student motivation?
(2) How these components affect the dimensions of student motivation?

We hypothesize that identifying the components of mobile AR applications that increase student motivation might contribute to inform the design and development of AR applications that effectively support student motivation.

To identify the components of a mobile AR application that increase student motivation, a research model constructed from the literature was empirically validated with data obtained from two sources: 35 students from four Vocational Education and Training (VET) institutes interacting with a mobile AR application called Paint-cAR^1^ for learning for a period of 20 days; and a self-report measure obtained from the Instructional Materials Motivation Survey (IMMS). The Paint-cAR application is a mobile AR application that was co-created with teachers, software developers and educational technology experts (Bacca, 2017). The Paint-cAR application is intended for teaching students about the process of repairing paint on a car which is one of the topics in the VET program of Car Maintenance. The Paint-cAR application was co-created with a Monitoring Module that automatically collects data about the interaction of students with the following modules of the application: a Scaffolding Module, a Real-time feedback Module, an Assessment Module and the AR Module.

Although AR technology has spread to almost all educational levels, and despite of the fact that AR has been extensively used for industrial maintenance, repair and assembly tasks (Lamberti et al., 2014; Bacca et al., 2015), there is still a lack of research exploring the benefits of this technology for VET education (Bacca et al., 2015). Consequently, we focus on this educational level to identify which are the components of a mobile AR application that might positively affect student motivation. On this educational level, motivational aspects are relevant for learning not only in the classroom, but also in the workplace (Schaap et al., 2012).

The contributions of this study are twofold: first, this study identifies some components of mobile AR applications that might positively affect student motivation in the VET level of education and second, this study presents the implications of these components and their associated variables on the design and development of motivational ARLEs.

This paper is organized as follows. After the introduction, the theoretical background that frames this paper is described followed by the related work. Then, the mobile AR application used in this study is described. The hypotheses development is then presented followed by the method. Then, hypotheses testing and results are presented followed by the implications of the study for the design and development of motivational mobile ARLEs and finally the limitations of this study are described.

## Theoretical Background

### Augmented Reality and Mobile Augmented Reality

The concept of AR was coined in contexts of training and maintenance when Caudell and Mizell (1992) introduced a Head-mounted display for assisting maintenance in the aircraft industry. Later, AR was defined as a technology that “allows the user to see the real world, with virtual objects superimposed upon or composited with the real world” (Azuma, 1997). Based on this definition and based on the definitions suggested by Dunleavy et al. (2009); Cuendet et al. (2013); Furió et al. (2013); Wu et al. (2013), we define AR as a technology that allows combining or annotating the real-time view of the physical world with any type of digital content generated by a computer or by a mobile device.

Kourouthanassis et al. (2015) defines Mobile Augmented Reality (MAR) as the systems that provide AR capabilities through mobile devices such as smartphones or tablets and differentiates MAR from the first generation of AR that is defined as Desktop Augmented Reality (DAR). In this paper we focus on MAR and marker-based AR.

### Motivation and the ARCS Model of Motivation

Motivation is a human dimension that explains why people make an effort to pursue a goal and why people actively work to attain that goal (Keller, 2010). While there are many models that study human motivation, one that explains this concept in relation to learning processes is the ARCS (*Attention*, *Relevance*, *Confidence*, and *Satisfaction*) model introduced by Keller (1987). The *attention* dimension refers to the interest of learners and their curiosity in the learning process. The *relevance* dimension refers to the learning process meeting the student’s learning needs and is related to the student’s perception on how the learning process is aligned with their own interests and goals. The *confidence* dimension relates to the opportunities that learners have to succeed in the learning activities. Finally, the *satisfaction* dimension is related to the feeling of success being reinforced and a sense of satisfaction with the results obtained in the learning process. The ARCS model has been used in previous studies that explore student motivation in ARLEs such as those conducted by Chin et al. (2015) and Chen et al. (2016).

In this paper, we draw on the ARCS model to represent student motivation in ARLEs with the aim to identify which is the components of ARLEs that positively affect student motivation.

### Universal Design for Learning (UDL)

The UDL is a validated framework that is based on neuroscience research for addressing students’ variability and avoids barriers in the learning process (Meyer et al., 2014). The UDL defines a set of principles that forms a practical framework for using technology to maximize learning opportunities for every student (Rose and Meyer, 2002). These three principles are: (1) Provide multiple means of engagement; (2) Provide multiple means of representation; and (3) Provide multiple means of action and expression. These principles are divided into guidelines and a wide variety of check points that provide recommendations on how to address students’ variability and avoid barriers in the learning process. These guidelines and checkpoints that are based on previous research provide insights into some aspects that have a positive impact on learning and motivation. In this paper we draw on the UDL framework together with the motivational design for learning theory to explain the relationship between some of the variables considered in this study (*use of scaffolding, real-time feedback, learning outcomes, degree of success, and time on-task*) with respect to student motivation. We draw on the UDL to explain how and why these variables might affect student motivation. In particular, we draw on the third UDL principle which is closely related to student motivation as one of the drivers of learning. Although the variables considered in this study do not come only from the UDL framework, the UDL framework is an important theoretical foundation that might provide us with insights into how some variables might have a positive effect on student motivation as one of the main aspects of expert learning.

## Related Work

### AR and Student Motivation

Research on AR in education has shown that, among many other advantages, AR experiences are useful for increasing student motivation when compared to non-AR experiences (Radu, 2014; Akçayır and Akçayır, 2017). Some studies have analyzed the impact of AR on student motivation using the ARCS model of motivation as summarized in **Table 1**. For each dimension, a (✓) indicates the dimensions in which AR had a remarkable effect and a (+) symbol indicates a positive effect but not remarkable.

**Table 1:** Studies that used the ARCS model to analyze the impact of AR on student motivation.

Study	Attention	Relevance	Confidence	Satisfaction	Learning domain/topic
Chen et al., 2016	+	+	✓	✓	Food chain (science)
Chiang et al., 2014	✓	✓	✓	+	Aquatic animals and plants (science)
Ibanez et al., 2015	✓	+	+	✓	Principles of electricity
Chen, 2013	✓	+	+	✓	Math
Di Serio et al., 2013	✓	+	+	✓	Italian renaissance art
Chin et al., 2015	+	+	+	✓	Liberal arts
Wei et al., 2015	+	+	+	+	Creative design teaching


Together these studies have used the ARCS model of motivation to represent the students’ levels of motivation. However, these studies do not clearly report which are the components of each AR application that positively affect the dimensions of the ARCS model of motivation. Consequently, it is still unclear how an AR application might affect student motivation. Apart from the ARCS model and the IMMS instrument, some researchers have used other questionnaires (and models) of motivation and they have found a positive impact of AR on student motivation. For instance, the study by Nachairit and Srisawasdi (2015) used the Scientific Motivation Questionnaire (SMQ), Martin-Gutierrez and Meneses (2014) used the R-SPQ-2F instrument. Other researchers have developed their own questionnaires to collect data about student motivation: Yin et al. (2013); Fonseca et al. (2014); Restivo et al. (2014); Laine et al. (2016). However, all of these studies also fall short in identifying the components of AR applications that might help to increase student motivation. According to Cheng and Tsai (2013), more research needs to be conducted in other dimensions of the learning experience such as motivation.

### Predictors of Student Motivation

Some studies report features, aspects or traits that might have impact on student motivation in ARLEs. **Table 2** shows these studies and the variables reported on each study.

**Table 2:** Studies that report variables that might impact on student motivation.

Author(s)	Variable - predictor (feature, aspect, trait, etc.)	Impact on student motivation
Ferrer et al., 2013	Usability	Despite the usability issues in mobile AR, student motivation can be improved.
Huang and Liaw, 2014	Immersion and interaction	Immersion and interactivity features are predictors of student motivation, but immersion is a stronger predictor.
Chen and Liao, 2015	Type of AR content (static and dynamic) Type of guiding strategies (procedure-guided or question-guided)	Learners in the static-AR and the procedure-guided strategy outperformed those learners in the dynamic-AR and the question-guided strategy in the dimension of intrinsic goal orientation
Chen and Wang, 2015	Learning styles	Learning styles do not affect learning motivation in mobile AR instruction.
Gopalan et al., 2016	Engagement Enjoyment Fun Ease of use	Engagement, Enjoyment and Fun were significant predictors of student motivation. Ease of use was not a predictor of motivation.


Overall, these studies provide insights into the variables that influence student motivation in ARLEs. However, these studies do not clearly report how these variables are connected with the components of AR applications and therefore it is not possible to determine which components of AR applications might produce a positive impact on student motivation. Thus, our study aims to contribute to the identification of the components of AR applications that might positively affect student motivation (modeled by the ARCS model of motivation) in ARLEs. We hypothesize that the identification of the components of AR applications that positively affect student motivation might help to inform the design and development of AR applications that effectively increase student motivation.

### The Mobile AR Application: Paint-cAR

Paint-cAR is a marker-based mobile AR application for supporting the teaching and learning process of repairing paint on a car in the context of the VET program on Car Maintenance. Repairing paint on a car is a complex process comprising a total of 30 steps divided into 6 phases (Cleaning, Sand down, Applying putties, Applying sealers, Painting, and Applying Clear Coats). Each phase has an average of five steps and each step in the process represents a task that needs to be done by using chemical products and/or tools to repair the paint. The steps must be done in a fixed order with respect to the other steps and only when all the steps in a phase are completed, that phase is completed and the next phase can start. In that regard, students need to learn how to perform each task (step in the process) and need to learn which are the chemical products and tools they need to use for each step in the process. Learning how to do this requires a considerable amount of time and combines theoretical and hands-on activities with chemical products and tools.

The Paint-cAR application was developed by the authors and is the result of a co-creation process, as described in the work by Bacca et al. (2015), in which VET teachers, software developers, and educational technology experts participated. Using the application, students learn about the chemical products and tools they need to use for each step of the paint repairing process. The application was developed with the following modules: a Scaffolding Module, a Real-time feedback Module, an Assessment Module, the AR Module, and a Monitoring Module. Furthermore, a booklet containing the AR markers that the application recognizes was given to students so that they can also use the application at home.

By using the application, students are guided through the process of repairing paint on a car step-by-step. For each one of the 30 steps, students must complete three activities that were designed by the VET teachers: (1) Watch a video that explains how experts go through the repairing process in that step. (2) Answer five multiple-choice questions about that step. (3) Identify the chemical products and/or tools they need to use for that step in the process. This last activity includes a mobile AR experience in which students need to move around the classroom (usually a workshop) and scan AR markers that are stuck to the tools and chemical products they need to use for that step in the process. The application recognizes if the product or tool is appropriate for a particular step in the process by identifying an ID associated to each marker.

**FIGURE 1:**
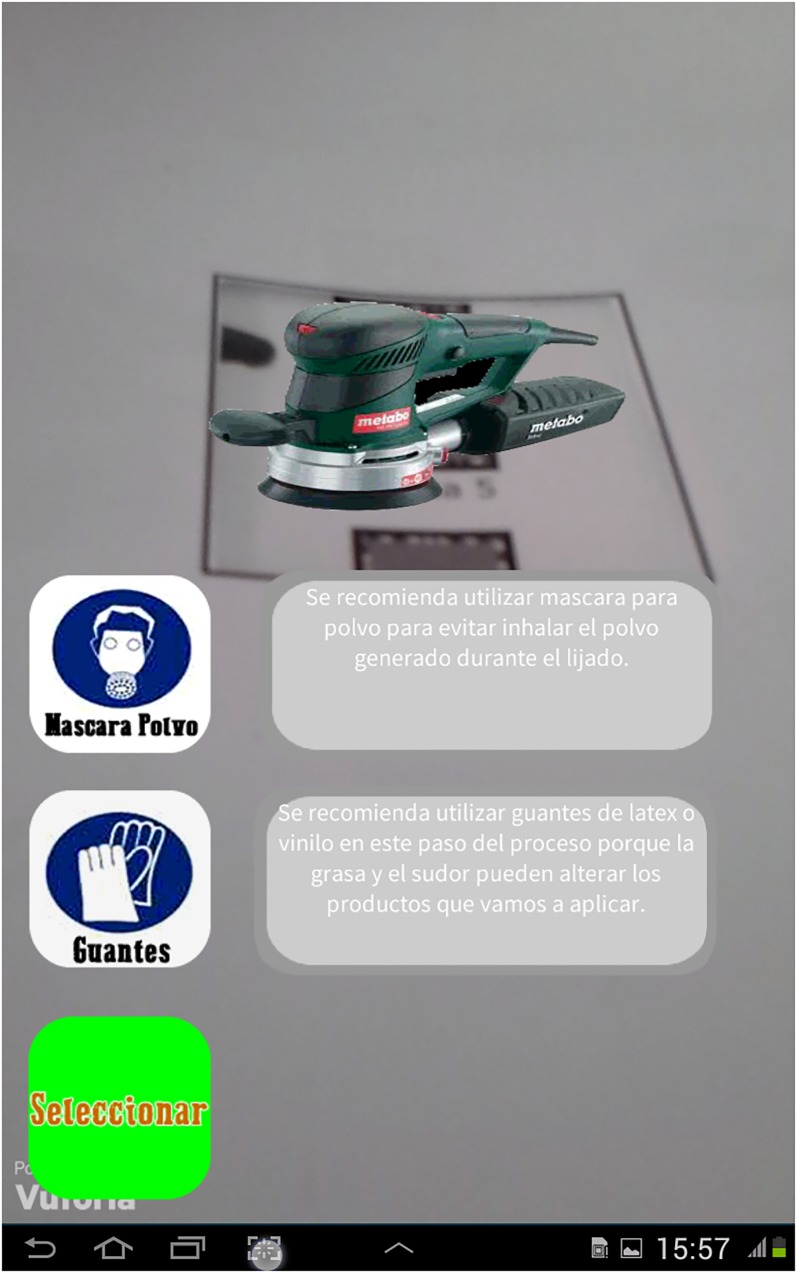
A screenshot of the Paint-cAR application in the AR mode.

In the AR experience, by using the Scaffolding Module students can ask the application for help at any time to obtain hints and information to help them to find the appropriate tools and chemical products in the workshop. The Real-time feedback Module provides feedback to students when they scan the markers stuck to the chemical products and tools so that students can reflect on their choices, successes and mistakes. The augmented information shown for each product and tool includes the characteristics of the product, the safety measures required when using it and its technical datasheet. Finally, the Monitoring Module captures students’ interaction with all the other modules. **Figure 1** shows a screenshot of the Paint-cAR application in the AR mode.

## Hypothesis Development

This study seeks to identify the components of an AR application that might positively affect student motivation. Thus, we defined five variables in total, two of them are associated to the interaction of the students with the scaffolding and feedback components of the AR application: *use of scaffolding and real-time feedback* and three of them are associated to students’ performance: *learning outcomes, degree of success*, and *time on-task*. In this study, we call this group of variables as the 5-VARLE (5 Variables of an ARLE). These variables are automatically measured by the Monitoring Module when students interact with the Paint-cAR application. These variables are not unique in ARLEs but they can appear in other learning experiences, such as web-based learning.

**Table 3** shows the modules of the Paint-cAR application with the 5-VARLE that are measured by the Monitoring Module and the associated module.

**Table 3:** Modules of the Paint-cAR application and the 5-VARLE.

Module in the application	Variable measured by the monitoring Module (the 5-VARLE)	Description of the variable	Events measured
Scaffolding Module	*Use of Scaffolding*	This variable represents the use of the Scaffolding Module in the Paint-cAR application during the ARLE.	The number of times that each student uses the Scaffolding Module.
Real-time feedback Module	*Real-time feedback*	This variable represents the use of the Real-time feedback Module when students interact with objects in the ARLE.	The number of times that each student read the feedback provided by the application in the AR experience. This event is registered when students do not ignore the message of feedback (close it).
Assessment Module	*Learning outcomes*	This variable represents the results of students when they answer the tests in the application.	A ratio of the number of test approved and the number of test answered in the application.
AR Module	*Degree of Success*	This variable represents the number of successful activities completed in the ARLE. This means when students succeed in selecting the correct product or tool they need to use for each step in the process of repairing paint on a car.	Number of correct products or tools selected in each one of the steps in the process of repairing paint on a car.
	*Time on-task*	This variable represents the amount of time that students spend in the ARLE using the Paint-cAR application.	The amount of time in seconds that students spend on the ARLE.


Based on the literature we theoretically defined the relationships of each one of the 5-VARLE with the four dimensions of the ARCS model of motivation to determine how the 5-VARLE might be related to student motivation. The purpose of this research model is to identify which variables support each dimension of motivation and therefore to determine which of these variables might be related to student motivation in an ARLE using a data-driven approach. This part of the study addresses the need expressed by Li et al. (2014) and Akçayır and Akçayır (2017) who claimed that more research is needed on the effect of AR on students’ motivation.

The relationship between scaffolding and motivation is established from a concept known as “success opportunities.” Success opportunities are the opportunities that learners have to succeed in tasks that are challenging (Keller, 2010). These opportunities might be different for students who have some basic knowledge and those who have more advanced knowledge. Scaffolding is a strategy that not only helps students to succeed in the activities in mobile ARLEs, but also creates success opportunities so that students can accomplish challenging tasks.

According to the UDL guidelines, graduated scaffolds are considered to be one of the key points for helping novice learners to reach mastery (Meyer et al., 2014). Moreover, according to Huang and Huang (2015) scaffolding has a positive effect on student motivation. In our study, we seek to determine if is there any relationship between the *use of scaffolding* and the four dimensions of the ARCS model of motivation. Therefore, the following hypothesis is suggested in which the independent variable is *use of scaffolding* and the dependent variables are the four dimensions of the ARCS model:

- **H_1_:** The *use of scaffolding* has a positive and significant correlation with the ARCS dimensions of motivation in mobile ARLEs.

As for the *real-time feedback*, some studies have reported that the provision of feedback might have a positive effect on student motivation. For instance, Chao et al. (2014) found that, providing specific feedback to students helps to motivate them. Likewise, in their literature review, Chakraborty and Muyia Nafukho (2014) found that one of the strategies for student engagement in distance learning is to provide consistent and timely feedback. Moreover, feedback is a key aspect in the *confidence* and *satisfaction* dimensions of motivation in the ARCS model (Keller, 2010). These studies suggest that *real-time feedback* might have a positive relationship with students’ motivation and the following hypothesis is suggested in which the independent variable is the *real-time feedback* and the dependent variables are the four dimensions of the ARCS model of motivation:

- **H_2_:** The provision of *real-time feedback* has a positive and significant correlation with the ARCS dimensions of motivation in mobile ARLEs.

*Degree of success* is another variable of the 5-VARLE. This variable represents the level at which students succeed in the learning activities. In other words it represents the student’s progress in the learning activities in the mobile AR experience. In this study we seek to identify if the *degree of success* might be correlated with student motivation in the VET level of education. The *degree of success* variable is closely related to the success opportunities. The students’ *degree of success* will increase if students are able to take advantage of the success opportunities taking into account the challenge imposed by the learning activity and their knowledge. The success opportunities are one the key aspects for supporting the *confidence* dimension of motivation (Keller, 2010). Consequently, we hypothesize that the students’ *degree of success* in an AR application positively affects the students’ levels of motivation during the intervention. Thus, the following hypothesis is suggested in which the independent variable is the *degree of success* and the dependent variables are the four dimensions of the ARCS model of motivation:

- **H_3_:** The students’ *degree of success* has a positive and significant correlation with the ARCS dimensions of motivation in mobile ARLEs.

As for the *learning outcomes* variable, it represents students’ achievement in the tests of the Assessment Module in the Paint-cAR application that evaluates the knowledge that students acquire during the mobile ARLE.

In the literature it is often reported that the students’ levels of motivation positively affect students’ achievement (*learning outcomes*) (Ai-Lim et al., 2010; Paechter et al., 2010; Castillo-Merino and Serradell-López, 2014; Ibanez et al., 2015; Eom and Ashill, 2016). Thus, according to the literature the following hypothesis is suggested in which the independent variables are the four dimensions of the ARCS model of motivation and the dependent variable is *learning outcomes*:

- **H_4_:** Student motivation (ARCS dimensions) has a positive and significant correlation with students’ *learning outcomes* in mobile ARLEs.

On the other hand, *time on-task* is considered to be one of the most important metrics of engagement and it has been used for the past 50 years (Ghergulescu and Muntean, 2016). *Time on-task* is also known as Academic Learning Time (ALT) which is the amount of time that students spend working on academic activities with the appropriate challenge for them (Berliner, 2007). ALT is also mediated by students’ engagement in the learning activity. Thus, the amount of time that students spend on learning activities is not the only factor that determines students’ learning outcomes. What really determines students’ learning outcomes is the ALT when students are engaged in the learning activities (Berliner, 2007).

However, to date little research has been done on the relationship between the *time on-task* or ALT and the use of AR in learning experiences. To the best of our knowledge only the study by Matcha and Awang Rambli (2015) has focused on studying the relationship between the *time on-task* and the use of an AR learning activity. The researchers analyzed the interaction of students in a collaborative AR activity about the basic concepts of electricity and concluded that on average 97% of the time students were focused on the learning activities showing the potential of AR for engagement in terms of the time spent on task.

Thus, in this study we seek to explore if the amount of time that students spend on the ARLE (*time on-task*) might have a relationship with student motivation. This might provide insights into the effect that the amount of time that students are exposed to an ARLE might increase or decrease their levels of motivation. In this context, the following hypothesis is suggested in which the independent variable is the *time on-task* and the dependent variables are the four dimensions of the ARCS model of motivation:

- **H_5_:** The amount of time that students spend in the ARLE (*time on-task*) has a positive and significant correlation with the ARCS dimensions of motivation in mobile ARLEs.

**FIGURE 2:**
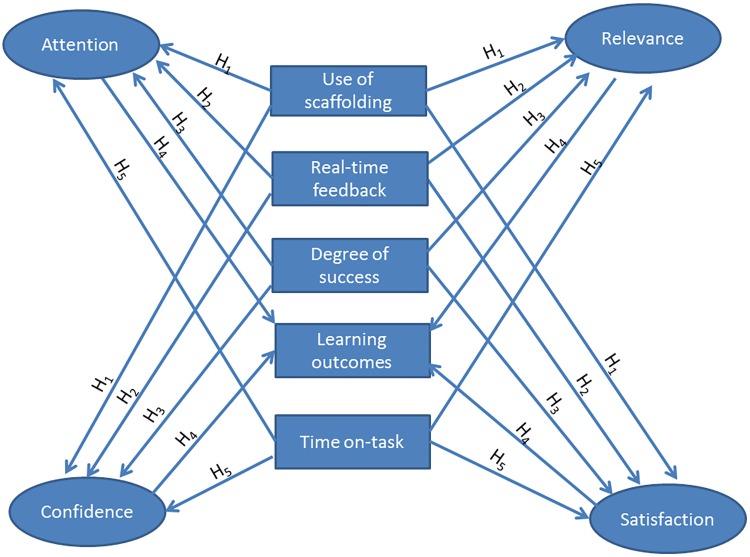
Research model for hypotheses H1, H2, H3, H4, and H5.

**Figure 2** shows the research model for hypotheses H_1_, H_2_, H_3_, H_4_, and H_5_. In short, this research model shows that the 5-VARLE is related to the four dimensions of the ARCS model of motivation.

## Method

Since the aim of this study is to identify the components of an AR application that might affect student motivation, the Paint-cAR application was used to collect data that was subsequently employed to validate the hypotheses defined. In this section the research design, participants and data collection methods are described. This study was carried out in accordance with the Ethical Guidelines for Educational Research (BERA). The protocol was approved by the Broadband Communications and Distributed systems group ethical committee from the University of Girona. All subjects gave written informed consent in accordance with the Declaration of Helsinki.

### Research Design and Participants

The Paint-cAR application was used to collect the data for this study. The application was used by 35 students enrolled in the intermediate training cycle “car bodywork” in the VET program of car maintenance. In this program, students learn about the process of repairing paint on a car. Students came from four VET institutes in Spain and had no previous knowledge about this topic before the intervention and the application complemented the traditional learning process. Students were not rewarded for their participation in this study and their participation did not have any implication for the grades in the subject matter. Before the intervention the 35 students answered a survey to collect demographic data and information about their experience using mobile technologies. The results of this survey provide an overview of the research sample in this study. As for the gender, 100% of the students were male. This result can be explained because the VET program of car’s maintenance is a program that mostly attracts male students. In the four VET institutes where we conducted the research it was not possible to include any woman in the research sample. 23% of the 35 students fall in the age range between 14–16 years old, 63% in the age range 17–19 years old, 9% in 20–22 years old, 3% in 23–25 years old, and 3% in more than 25 years old.

As for their experience with mobile devices, 100% of the students had their own smartphone and 91% of them had internet in their smartphone. The type of internet connection that students used at the time of the intervention was: 66% used 4G, 57% used the Wi-Fi of the VET institute, and 31% used 3G. We also asked students about the activities they do with the mobile devices. For this question students could mark multiple options. Students reported that they used the mobile device for the following purposes: Chat with friends (97%), Make calls (97%), Check email (83%), Social networking (83%), Sail on the internet (77%), Use GPS (57%), and play videogames (46%). Students reported that they install applications once a week (37%), almost every day (17%) and once a month (26%), and almost never (20%). Finally, students reported that 63% of them usually use a laptop for doing the homework and that 71% of them have used mobile educational applications in their smartphone. Moreover, 97% of the students reported that they would like to use mobile educational applications for learning in the VET program.

As for the experimental mortality, at the beginning of the research study 63 students were invited to take part in this study. However, before starting the study 15 students were not able to participate because their mobile devices were not compatible with the Paint-cAR application. The study began with 48 students but after the first week of the experiment five students bought a new mobile device or their mobile device was broken and therefore they had to install the application again. This invalidated the data coming from these students. Finally, eight students drop out from the study because they prefer to work in the real workshop instead of using the Paint-cAR application.

Students used the application for 20 days as a support for the learning process. During this time, the Monitoring Module captured the interaction of students with the modules of the Paint-cAR application as they learned with the application. The procedure for the intervention was as follows:

- **Application installation:** In this phase, students were guided in the process of downloading and installing the application. Students were then shown how to use the application. A booklet containing the markers the application recognizes was given to each student so they could use the application at home. This phase lasted for 60 min.
- **Use of Paint-cAR application for learning (I):** Students used the application either at home or in the classroom (usually a workshop in the VET institution) to learn about the process of repairing paint on a car. This phase lasted for 1 week.
- **First test in the workshop:** One week after students had been using the application, a class test in the classroom (workshop) was carried out. The markers that the application recognizes were placed in the corresponding locations around the workshop and students then had to find the appropriate tools or chemical products to complete the process of repairing paint on the hood of a car. The test lasted (on average) 2 h for each VET institute. At the end of the test, students received feedback from the teachers on their performance in the test.
- **Use of Paint-cAR application for learning (II):** In this phase, students used the application to learn at home (using the booklet) or in the workshop. Teachers advised each student to use the application for practicing about the topics in which they found more difficulties. The learning process was supported by the teachers in class. This phase lasted for 1 week and a half.
- **Second test in the workshop:** After the period of practicing with the application at home and in the workshop, a second test in was carried out in the workshop. The test lasted an average of 2 h for each VET institution.
- **Instructional Materials Motivation Survey Motivation instrument:** Following the second test in the workshop, the IMMS instrument (Keller, 2010) was applied to gather information about the levels of motivation in the four dimensions of the ARCS model with respect to the use of the application.

### Data Sources and Data Collection

Studies exploring student motivation in ARLEs, typically use self-report measures of validated questionnaires such as the IMMS (Keller, 2010) or other instruments such as the SMQ, or tailor-made questionnaires. However, self-report measures have some disadvantages. For instance, some instruments do not provide the level of detail needed by the researcher to interpret the results. In other cases, some experiences are unconscious and cannot be fully expressed by people using a questionnaire (Barker et al., 2002). Consequently, there might be some issues of validity in the results obtained only from self-report measures. Thus, it is recommended to supplement self-report data with other sources of information like observational data (Barker et al., 2002) or to collect more information during the learning experience instead of doing it at the beginning or at the end (Fonseca et al., 2014). Hence, in this study we supplemented the self-reported measure (the IMMS instrument) with the automatic Monitoring Module developed in the application that collected all the interaction of students with the application.

As mentioned earlier, the Paint-cAR application was developed with a Monitoring Module that continuously tracks student interaction with the modules of the Paint-cAR application. **Table 4** shows the modules of the Paint-cAR application together with the description of the events tracked, data captured for each event and the number of events tracked.

**Table 4:** Events measured in the 5-VARLE.

Variable measured by the Monitoring Module (the 5-VARLE)	Description of events tracked	Number of events tracked
*Use of Scaffolding*	Registers the number of times that each student uses the Scaffolding Module during the ARLE.	1150
*Real-time feedback*	The variable registers the exact moment when students received feedback after a mistake is made during the completion of a learning activity in the AR experience.	766
*Degree of success*	The variable registers when students successfully complete a task in the ARLE. This includes selecting the appropriate products or tools to use for each step in the repairing process.	852
*Learning outcomes*	This variable stores a ratio of the number of test approved and the number of test answered in the application. Each test is managed by the Assessment Module and consists of five multiple-choice questions. The questions for each test are randomly selected from a database of 109 questions classified by topic and created by the teacher. The test is approved with the five correct answers.	309
*Time on-task*	This variable registers the amount of time that students spend on the ARLE.	7781


Information registered by the Monitoring Module in the student’s smartphone or tablet is sent to a server with an ID of the student that interacted with the application. In total 10.858 events of interaction for all the students were detected by the Monitoring Module and sent to the server during the 20 days of the intervention. Thereby, we collected data from the interaction of students with the application and we used this data to validate the research models that we described in section 4.

As for manual data sources, the IMMS instrument (Keller, 2010) was used to gather information about students’ motivation at the end of the intervention. The IMMS instrument has a total internal consistency (Cronbach alpha) of 0.95. We also estimated the internal consistency reliability of the instrument with the data we collected and the overall result for the IMMS instrument was: 0.82. Moreover, for each dimension of motivation the results were: Attention (0.86), Relevance (0.66), Confidence (0.6), and Satisfaction (0.73). These results show that the reliability of the instrument was between the medium to high levels.

## Hypotheses Testing and Results

By testing the hypotheses in the research model defined earlier in section 4 (see **Figure 2**), the variables correlated with student motivation can be identified to obtain an empirical model of student motivation in mobile ARLEs.

To determine the relationships between the 5-VARLE and the four dimensions of the ARCS model of motivation from an exploratory perspective, Spearman’s rho correlations were applied because data collected do not follow a normal distribution. Correlation has also been used in hypothesis validation in other studies conducted by Chou et al. (2010); Sylaiou et al. (2010); Bulu (2012) to show the association between variables but not to explain the causality between them. However, correlation may provide insights between the associations of some variables in a model. Coolican (2014) states that correlations are useful in providing evidence that supports a theory and form part of the evidence in many theories in social science. In this section, the results of the hypotheses validation are described and organized according to each hypothesis.

**FIGURE 3:**
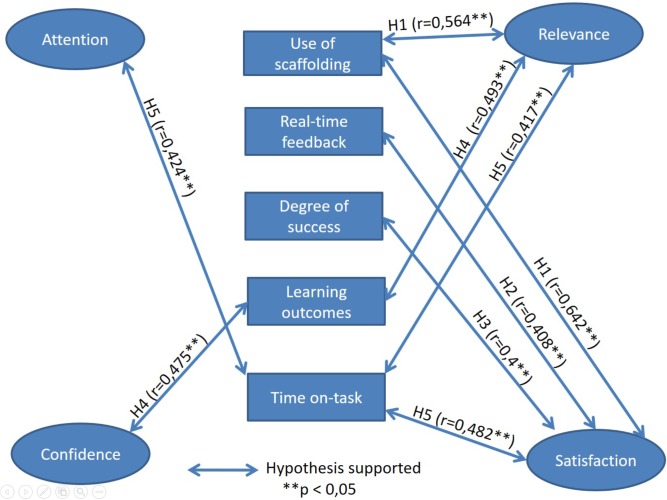
Validated research model for hypotheses H1, H2, H3, H4, and H5.

To identify if data followed a normal distribution for each hypothesis, the values of data skewness and kurtosis used for validating the hypothesis were analyzed together with the Kolmogorov-Smirnov and Shapiro-Wilk tests for normality. **Table 5** summarizes the results of the correlations between the dimensions of the ARCS model and the variables in hypothesis H_1_, H_2_, H_3_, H_4_, and H_5_. This table includes the degrees of freedom for each correlation. **Figure 3** shows the validated research model for hypothesis H_1_, H_2_, H_3_, H_4_, and H_5_.

**Table 5:** Correlation between the dimensions of the ARCS model and the variables in hypothesis H_1_, H_2_, H_3_, H_4_, and H_5_.

	Use of Scaffolding (H_1_)	Real-time feedback (H_2_)	Degree of success (H_3_)	Learning outcomes (H_4_)	Time on-task (H_5_)
Attention	—	—	—	—	*r* = 0.424 ^∗∗^(df = 29)
Relevance	*r* = 0.564 ^∗∗^(df = 34)	—	—	*r* = 0.493 ^∗∗^(df = 34)	*r* = 0.417 ^∗∗^(df = 29)
Confidence	—	—	—	*r* = 0.475 ^∗∗^(df = 34)	—
Satisfaction	*r* = 0.642 ^∗∗^(df = 34)	*r* = 0.408 ^∗∗^(df = 34)	*r* = 0.4 ^∗∗^(df = 29)	—	*r* = 0.482 ^∗∗^(df = 29)


*^∗^*p* < 0.1; ^∗∗^*p* < 0.05; ^∗∗∗^*p* < 0.001; df = degrees of freedom.*

### Hypothesis Regarding the Use of Scaffolding

For hypothesis H_1_, a Spearman correlation on the *use of scaffolding* strategy and the four dimensions of the ARCS model was used. There is a positive moderate correlation between the *use of scaffolding* and the *relevance* (*r* = 0.564, *p* = 0.012) and the *satisfaction* dimensions of motivation (*r* = 0.642, *p* = 0.003). However, no significant relationship was found between the *use of scaffolding* and the *attention* and *confidence* dimensions (see **Table 5** and **Figure 3**).

The results show that the *use of scaffolding* supports the *relevance* and *satisfaction* dimensions of motivation in mobile ARLEs. This result may be explained by the fact that *use of scaffolding* helps students to complete the learning tasks, which means that it provides the resources, such as information or instructions that students need to accomplish the task. In that regard, according to the UDL guidelines, if the learning environment provides the appropriate challenging tasks along with the resources to complete those tasks, students will be able to find the tasks that are motivating for them (Meyer et al., 2014). Our results, are in line with the results obtained by Chen et al. (2016) who found that scaffolding in AR applications supported the *confidence* and *satisfaction* dimensions.

We confirmed that providing scaffolds in mobile ARLEs in the VET level of education may help to create in students a positive view and perception of the learning task because the scaffolds help students to accomplish the task and reduce the levels of frustration and/or discouragement.

### Hypothesis Regarding the Real-Time Feedback

For hypothesis H_2_, a Spearman correlation on the use of *real-time feedback* and the four dimensions of the ARCS model showed that there is a positive and significant moderate correlation between the feedback provided to the student in the AR activities and the *satisfaction* dimension of motivation (*r* = 0.408, *p* = 0.021). See **Table 5** and **Figure 3**.

The results show a relationship between *real-time feedback* and the *satisfaction* dimension of motivation. One of the strategies for promoting feelings of *satisfaction* is “intrinsic reinforcement” (Keller, 2010). This implies using positive feedback to reinforce students’ positive feelings to improve *satisfaction*. These results are also in line with the recommendations of the UDL with respect to providing feedback that encourage perseverance (Meyer et al., 2014). The UDL recommends providing mastery-oriented feedback, i.e., feedback that helps students to reach mastery, rather than simply confirming their success or pointing out errors. Moreover, Chakraborty and Muyia Nafukho (2014) found that one of the strategies for engagement in distance learning is to provide timely and consistent feedback. Thus, the provision of timely and consistent feedback in mobile ARLEs might help to increase *satisfaction*. To the best of our knowledge, little research has previously evaluated the effect of *real-time feedback* on student motivation in mobile ARLEs. Consequently, our results contribute to the knowledge on the effect that *real-time feedback* has on student motivation in mobile ARLEs in VET. However, further studies should explore the relationship between *real-time feedback* and the *attention*, *relevance*, and *confidence* dimensions in mobile ARLEs in other educational levels.

### Hypothesis Regarding the Degree of Success

In H_3,_ a Spearman correlation on the students’ *degree of success* and the four dimensions of the ARCS model showed that there is a positive moderate and significant correlation between the students’ *degree of success* and the *satisfaction* dimensions of motivation (*r* = 0.4, *p* = 0.029) (See **Table 5** and **Figure 3**). The correlations with the other dimensions of motivation (*attention*, *relevance*, and *confidence*), were not significant.

The results show a relationship between the student’s *degree of success* and the *satisfaction* dimension. As mentioned earlier, the *degree of success* is related to the success opportunities and therefore is related to the *confidence* dimension of motivation. However, we found that in the VET level of education the *degree of success* seems to be more related to the *satisfaction* dimension of motivation. One possible explanation of this result is that student *success opportunities* supported by the *use of scaffolding* and *real-time feedback* seem to be a rewarding experience for the students and therefore *satisfaction* also increases. According to Keller (2010), rewarding outcomes form one of the strategies for promoting feelings of *satisfaction*. This means that in mobile ARLEs in VET, completing challenging tasks with the support of scaffolding and real-time feedback is a rewarding outcome for students and that will increase their *satisfaction*. Our results contribute to the understanding of the relationship between the students’ *degree of success* and the *satisfaction* dimension as well as the implications of the modules needed to support the students’ *degree of success* (scaffolding and real-time feedback). Our results are in line with the study by Cabero et al. (2017) who found a moderate correlation between students’ degree of success and the four dimensions of the ARCS model of motivation.

### Hypothesis Regarding Learning Outcomes

As for hypothesis H_4_, a Spearman correlation on the students’ *learning outcomes* and the four dimensions of the ARCS model showed that there is a positive and significant moderate correlation between the students’ *learning outcomes* and the *relevance* (*r* = 0.493, *p* = 0.023) and *confidence* (*r* = 0.475, *p* = 0.029) dimensions of motivation. These results are summarized in **Table 5** and **Figure 3**.

Keller (2010), on the one hand, highlights three strategies for supporting *relevance*: goal orientation, motive matching and familiarity. If the mobile ARLE support these strategies, the *relevance* dimension of motivation might be increased. On the other hand, in terms of *confidence*, the results agree with the findings of Hsieh (2014), who found that students with higher levels of *confidence* and with an expectance of success report better *learning outcomes*. In general, we confirmed that learning motivation is related to students’ *learning outcomes* in AR experiences in VET. Other researchers have found similar results in other educational contexts (Paechter et al., 2010; Castillo-Merino and Serradell-López, 2014; Eom and Ashill, 2016; Cabero et al., 2017; Tsai et al., 2017).

### Hypothesis Regarding the Time On-Task

As for hypothesis H_5,_ a Spearman correlation on the students’ *time on-task* and the four dimensions of the ARCS model of motivation showed that there is a positive moderate and significant correlation between the *time on-task* and *attention* dimension (*r* = 0.424, *p* = 0.024). Besides that, a positive moderate and significant correlation was found between *time on-task* and *relevance* dimension (*r* = 0.417, *p* = 0.027). Finally, a positive moderate and significant correlation was found between *time on-task* and *satisfaction* dimension (*r* = 0.482, *p* = 0.009). These results are summarized in **Table 5** and **Figure 3**.

Interestingly, the student *time on-task* has a moderate correlation with the *attention*, *relevance*, and *satisfaction* dimensions. Keller (2010) states that *time on-task* is a direct measure of motivation. From this perspective, the overall results suggest that if students spend more time on the mobile ARLE, their levels of motivation in the dimensions of *attention*, *relevance*, and *satisfaction* might increase. This result might confirm that AR has the potential to increase students’ *time on-task* and therefore increase student motivation.

No significant correlation was found between the *time on-task* and the *confidence* dimension of motivation. This result may be explained by the fact that an increase in the time spent on a learning activity is not perceived as positive. The amount of time that someone spends on learning activities is often perceived as a measure of the skills or abilities that the person has for solving the problem or completing the activity. Thus, *confidence* may be decreased because of a negative perception in terms of a lack of ability to solve the problem. This feeling may be stronger in a group in which some students might perceive that their classmates are solving the problems more quickly than them. Hence, *confidence* dimension may be decreased. However, this claim needs to be analyzed in future studies.

## Implications of this Study for the Design of Motivational Mobile AR Learning Experiences

This section summarizes the main implications and findings obtained from the hypotheses validation and we provide some recommendations on the design of motivational mobile ARLEs for the VET level of education. Since these implications have been obtained from a marker-based mobile ARLE, the recommendations provided are intended for the design and development of marker-based mobile AR. However, some of the recommendations could be extended to desktop AR and other types of AR but further research is needed to confirm if the recommendations are valid for other types of AR or for other educational levels.

In this study, we found insights into the relationships that might exist between the 5-VARLE and the dimensions in the ARCS model of motivation.

The *use of scaffolding* was found to be related to the *relevance* dimension and *satisfaction* dimension of motivation (see hypothesis H_1_). This finding suggests that mobile ARLEs for the VET level of education might include a Scaffolding Module to support the *relevance* and *satisfaction* dimensions of motivation. Based on the main characteristics of the *relevance* dimension in the ARCS model of motivation, the Scaffolding Module would need to be designed in a way that helps to create a positive perception of the learning task in terms of usefulness and meaningfulness (Keller, 2010) so that students can feel that the learning task is connected to their life and personal needs or interests. On the other hand, the Scaffolding Module should be designed to provide positive reinforcement and effective assistance to students to achieve in the learning task. In short, the Scaffolding Module needs to provide relevant information at the appropriate time and in the appropriate format to assist students in the learning task.

*Real-time feedback* in mobile ARLEs was found to be related to the *satisfaction* dimension of motivation (see hypothesis H_2_) in mobile ARLEs. Hence, we recommend that *real-time feedback* should provide intrinsic reinforcement, meaning that feedback should be positive and reinforce students’ feelings of achievement and engagement (Keller, 2010) throughout the task. This means that the feedback needs to guide students in the ARLE and provide meaningful information at the right time rather than just confirming their success or rather than just indicating mistakes during the experience. The amount of information in the real-time feedback Module needs to be adjusted to the students’ needs and context.

As for the *degree of success*, this variable was also found to be associated to the *satisfaction* dimension of motivation (see hypothesis H_3_). This might suggest that a mobile AR application for the VET level of education which allows students to succeed in challenging tasks might help to promote a positive perception of satisfaction with the learning experience. Although the relationship between *degree of success* and *satisfaction* might be present in other learning experiences different from AR, our findings confirm that student’s *degree of success* is also a factor that positively affects motivation in ARLEs.

We also found that in ARLEs, the students’ *learning outcomes* are highly related to the *relevance* dimension and *confidence* dimension of motivation (see hypothesis H_4_). As for the *relevance* dimension, Keller (2010) suggest that the learning content needs to be aligned with the students’ needs and interests and should be connected with their life experiences so that it can be relevant. For the *Confidence* dimension, Keller (2010) recommends providing the success opportunities and personal control. The success opportunities can be created with scaffolding by adjusting the appropriate level of challenge for each student’s needs and preferences and providing the right information in the right moment. As for personal control, the real-time feedback should be mastery-oriented (Meyer et al., 2014) and positive attributional (Keller, 2010).

We also found that the *time on-task* variable in mobile ARLEs is moderately related to the *attention*, *relevance*, and *satisfaction* dimensions of motivation (see hypothesis H_5_). This means that the amount of time that students spend on the ARLE is highly related to the *attention*, *relevance*, and *satisfaction* dimensions of motivation. Although it is generally recognized that student motivation is needed to increase student *time on-task*, in this study we sought to identify if the amount of time that students spend on the ARLE might have any effect on student motivation. This finding provides insights into the effect that the time that students spend on the ARLE might have on student motivation. In particular, our findings might suggest that, those mobile AR applications that are able to capture the interest of the students and increase their time on the learning activities, are the applications that better support student motivation. However, further research is needed in other educational levels and with other types of AR to validate this claim.

## Limitations of this Study

The study was conducted in only one VET program (VET program of Car’s Maintenance) and this might limit the scope of some of the findings to that VET program. Moreover, the Paint-cAR application is a marker-based AR application and therefore the results obtained in this study might not apply to other types of AR reality, such as marker-less or location-based AR. Consequently, the results need to be interpreted with some caution.

Another potential limitation is that we only considered a group of variables represented in the 5-VARLE but other variables might be included in similar studies to uncover new relationships.

## Ethical Considerations

This study was carried out in accordance with the Ethical Guidelines for Educational Research (BERA). The protocol was approved by the Broadband Communications and Distributed systems group ethical committee from the University of Girona. All subjects gave written informed consent in accordance with the Declaration of Helsinki.

## Author Contributions

JB designed and developed the AR application and conducted the tests with students in real settings. SB conducted statistical analysis and the validation of hypotheses. RF collected bibliography for the related work, theoretical background and wrote these sections in the paper. K wrote the section of the paper about the implications of this study for the design and development of motivational AR learning experiences and contributed to the discussion section.

## Conflict of Interest Statement

The authors declare that the research was conducted in the absence of any commercial or financial relationships that could be construed as a potential conflict of interest.

## Footnotes

1 http://piranya.udg.edu/pintuRA/
